# Structure of Germline Immunoglobulin Heavy-Chain γ1 Transcripts in Interleukin 4 Treated Mouse Spleen Cells

**DOI:** 10.1155/1990/47659

**Published:** 1990

**Authors:** Minzhen Xu, Janet Stavnezer

**Affiliations:** Department of Molecular Genetics and Microbiology University of Massachusetts Medical Center 55 Lake Avenue North Worcester Massachusetts 01655 USA

## Abstract

Antibody class switching is mediated by a DNA recombination event that replaces the Cμ gene with one of the other heavy (H) chain constant region (C_H_) genes located 3' to the Cμ gene. The regulation of this process is essential to the immune response because different C_H_ regions provide different biological functions. Correlative evidence indicates that the isotype (class) specificity of the switch is determined by the accessibility of specific C_H_ genes as indicated by hypomethylation and transcriptional activity. For example, RNAs transcribed from specific unrearranged C_H_ genes are induced prior to switching under conditions that promote subsequent switching to these same C_H_ genes. The function of transcription of these germline C_H_ genes is unknown. In this report, we describe the structure of RNA transcribed from unrearranged γ1 genes in mouse spleen cells treated with LPS plus .a HeLa cell supernatant containing recombinant interleukin 4. The germline γl RNA is initiated at multiple start sites 5' to the tandem repeats of the γ1 switch (Sγ1) region. As is true for analogous RNAs transcribed from unrearranged γ2b and c genes, the germline γ1 RNA has an exon transcribed from the region 5' to Sγ1 sequences, which is spliced at a unique site to the Cr gene. The germline γ1 RNA has an open-reading frame (ORF) that potentially encodes a small protein 48 amino acid in length.


*Developmental Immunology*, 1990, Vol. 1, pp. 11–17
Reprints available directly from the publisher
Photocopying permitted by license only

© 1990 Harwood Academic Publishers GmbH
Printed in the United Kingdom

### Structure of Germline Immunoglobulin Heavy-Chain $\gamma 1$ Transcripts in Interleukin 4 Treated Mouse Spleen Cells

MINZHEN XU* and JANET STAVNEZER

*Department of Molecular Genetics and Microbiology, University of Massachusetts, Medical Center, 55 Lake Avenue North, Worcester, Massachusetts 01655*

Antibody class switching is mediated by a DNA recombination event that replaces the $C_\mu$ gene with one of the other heavy (H) chain constant region ($C_H$) genes located 3′ to the $C_\mu$ gene. The regulation of this process is essential to the immune response because different $C_H$ regions provide different biological functions. Correlative evidence indicates that the isotype (class) specificity of the switch is determined by the accessibility of specific $C_H$ genes as indicated by hypomethylation and transcriptional activity. For example, RNAs transcribed from specific unrearranged $C_H$ genes are induced prior to switching under conditions that promote subsequent switching to these same $C_H$ genes. The function of transcription of these germline $C_H$ genes is unknown. In this report, we describe the structure of RNA transcribed from unrearranged $\gamma 1$ genes in mouse spleen cells treated with LPS plus a HeLa cell supernatant containing recombinant interleukin 4. The germline $\gamma 1$ RNA is initiated at multiple start sites 5′ to the tandem repeats of the $\gamma 1$ switch ($S_{\gamma 1}$) region. As is true for analogous RNAs transcribed from unrearranged $\gamma 2b$ and $\alpha$ genes, the germline $\gamma 1$ RNA has an I exon transcribed from the region 5′ to $S_{\gamma 1}$ sequences, which is spliced at a unique site to the $C_\gamma$ gene. The germline $\gamma 1$ RNA has an open-reading frame (ORF) that potentially encodes a small protein 48 amino acid in length.

KEYWORDS: I exon, immunoglobulin class switching, interleukin 4, polymerase chain reaction, recombination.

### INTRODUCTION

A functional immunoglobulin (Ig) heavy (H) chain gene is comprised of several gene segments that are brought together by gene rearrangement during differentiation of antibody-producing cells. The antigen binding site is encoded by variable (V), diversity (D), and joining (J) segments that are initially associated with the $C_\mu$ constant region gene. After immunization, the same VDJ gene may subsequently be expressed with other downstream H-chain constant ($C_H$) region genes by a process called class switching, thus changing the effector function of the antibody while maintaining its antigen specificity. Class switching occurs by a DNA recombination mediated by repetitive sequences known as the switch (S) regions that lie a few kilobases upstream of each $C_H$ gene (except $C_\delta$). A VDJ gene initially associated with the $C_\mu$ gene can be translocated to a downstream $C_H$ gene by switch recombination occurring between the $S_\mu$ region and the S region of a target $C_H$ gene (reviewed in Grimacher, in press).

Aspects of the process of class switching can be studied in cultured cells. Normal $IgM^+$ splenic B cells from mice will switch to $IgG_3$ and $IgG_{2b}$ expression after polyclonal stimulation in culture with lipopolysaccharide (LPS) (Bergstedt-Lindqvist et al., 1984). T-cell-derived lymphokines can influence the isotype to which the cells switch (Isakson et al., 1982; Bergstedt-Lindqvist et al., 1984, 1988; Coffman et al., 1988; Lebman and Coffman, 1988). The addition of interleukin 4 (IL-4) to lipopolysaccharide (LPS) induced spleen B cells stimulates switching to $IgG_1$ and IgE and suppresses switching to $IgG_3$ and $IgG_{2b}$. Several lines of evidence indicate that IL-4 directs the switch recombination to the $\gamma 1$ and $\varepsilon$ genes. For example, IL-4 has been shown to increase the frequency of precursors for $IgG_1^+$ and $IgE^+$ cells

*Corresponding author.

(Bergstedt-Lindqvist et al., 1988; Coffman et al., 1988; Lebman and Coffman, 1988). IL-4 appears to direct the switch to $\gamma 1$ and $\varepsilon$ by increasing the accessibility of the $\gamma 1$ and $\varepsilon$ genes, as shown by the fact that IL-4 induces RNAs transcribed from unrearranged $\gamma 1$ and $\varepsilon$ genes and reduces the level of RNA transcribed from unrearranged $\gamma 2b$ genes prior to the expression of $IgG_1$ and IgE (Lutzker et al., 1988; Rothman et al., 1988; Stavnezer et al., 1988; Berton et al., 1989; Esser and Radbruch, 1989). Although the function of transcription of the germline $C_H$ genes is unknown, strong correlative evidence indicates that only unrearranged $C_H$ genes that are transcriptionally active are capable of undergoing switch recombination (Stavnezer-Nordgren and Sirlin, 1986; Yancopoulos et al., 1986; Lutzker et al., 1988; Rothman et al., 1988; Stavnezer et al., 1988; Berton et al., 1989; Esser and Radbruch, 1989; Severinson et al., in press). RNA transcription may simply be a by-product of the accessibility of $C_H$ genes to switch recombinase. Alternatively, transcription might serve as part of the mechanism of switch recombination or the transcripts or a polypeptide encoded by them might direct class switching. To identify DNA sequences necessary for their regulation and to understand the possible function(s) of germline transcripts, it is first necessary to know the structure of the germline transcripts. We report here the structure of $\gamma 1$ germline transcripts induced in mouse spleen cells by treatment with LPS plus a HeLa cell supernatant containing rIL-4. We find that the structure of the $\gamma 1$ germline RNA is similar to that of RNAs transcribed from unrearranged $C_{\gamma 2b}$ and $C_\alpha$ genes (Lutzker and Alt, 1988; Radcliffe et al., 1990).

### RESULTS

### LPS and IL-4 Induce Germline $\gamma 1$ RNA Transcripts

We have previously shown that treatment of spleen cells with IL-4 or a supernatant from a $T_{H2}$ cell line that contains IL-4 (Noma et al. 1986) (and other factors) in the presence or absence of LPS induces in B cells transcripts from unrearranged $C_{\gamma 1}$ genes that hybridize with the $5'S_{\gamma 1}$ HindIII-PstI segment (Fig. 1A) as 1.7-kb and 3.2-kb RNA species (Stavnezer et al., 1988; Severinson et al., in press). We began to localize the sequences encoding the I exon of germline $\gamma 1$ RNA by additional RNA blotting experiments. The 2.7-kb HindIII-PstI fragment derived from clone p$\gamma$1/EH10 (Mowatt and Dunnick, 1986) was subcloned into three fragments: a 1.2-kb HindIII-BamHI fragment, a 0.8-kb BamHI-KpnI fragment, and a 0.7-kb KpnI-PstI fragment (Fig.1A). Labeled RNA probes transcribed from these fragments were hybridized with blots containing poly(A)+ RNA from mouse spleen cells induced for 2 days with LPS and IL-4. Of these three fragments, only the KpnI-PstI probe detected the 1.7-kb and 3.2-kb RNAs (Fig. 1B). No RNA was detected by the HindIII-BamHI or BamHI-KpnI probes (data not shown). No RNA was detected with a KpnI-PstI probe for antisense transcripts (data not shown). This result indicates that the I exon of germline $\gamma 1$ RNA is encoded within the KpnI-PstI fragment.

### Determination of Splice Site of Germline $\gamma 1$ RNA

In order to precisely locate the $I_{\gamma 1}$ exon, we used the PCR to prepare cDNA clones containing the 3′ donor splice site of the $I_{\gamma 1}$ exon. Based on the RNA blotting data described before and previous work (Stavnezer et al., 1988), we expected that the germline $\gamma 1$ RNAs were initiated within the KpnI-PstI fragment and spliced to the $C_{\gamma 1}$ gene. To show this, we obtained a $C_{\gamma 1}$ oligonucleotide complementary to the 5′ end of the $C_{\gamma 1}$ gene (Figs. 1A and 2C) and an oligonucleotide (oligo 4) containing sequences located from 448 to 465 nucleotides 3′ of the KpnI site (Figs. 1A and 2A). Oligo 4 primes DNA synthesis toward the 3′ direction and the $C_{\gamma 1}$ oligo primes DNA synthesis toward the 5′ direction. Using the PCR to amplify cDNA products from poly(A)+ RNA from spleen cells treated for 2 days with LPS and 15% rIL-4-containing HeLa cell supernatant, we obtained several cDNA clones that should contain the splice site of germline $\gamma 1$ RNA. Eight of these clones were sequenced. All eight of these clones had the identical splice donor located between nucleotides 633 and 634 in Fig. 2A and demonstrated that the splice acceptor at the 5′ end of the $C_{\gamma 1}$ gene, which is used in $\gamma 1$ mRNA, is used for germline $\gamma 1$ RNA. The location of the $I_{\gamma 1}$ splice donor is consistent with RNase protection experiments in which a predominant protected band of 281 bp was obtained after hybridization of total cell RNA with a RNA probe transcribed from the BglII-PstI segment (data not shown). These results are also consistent with S1 protection experiments of Berton et al. (1989). The splice junction of the $I_{\gamma 1}$ and $C_{\gamma 1}$ exons employ consensus donor and acceptor sequences (Figs. 2A and 2B) (Lewin, 1980).

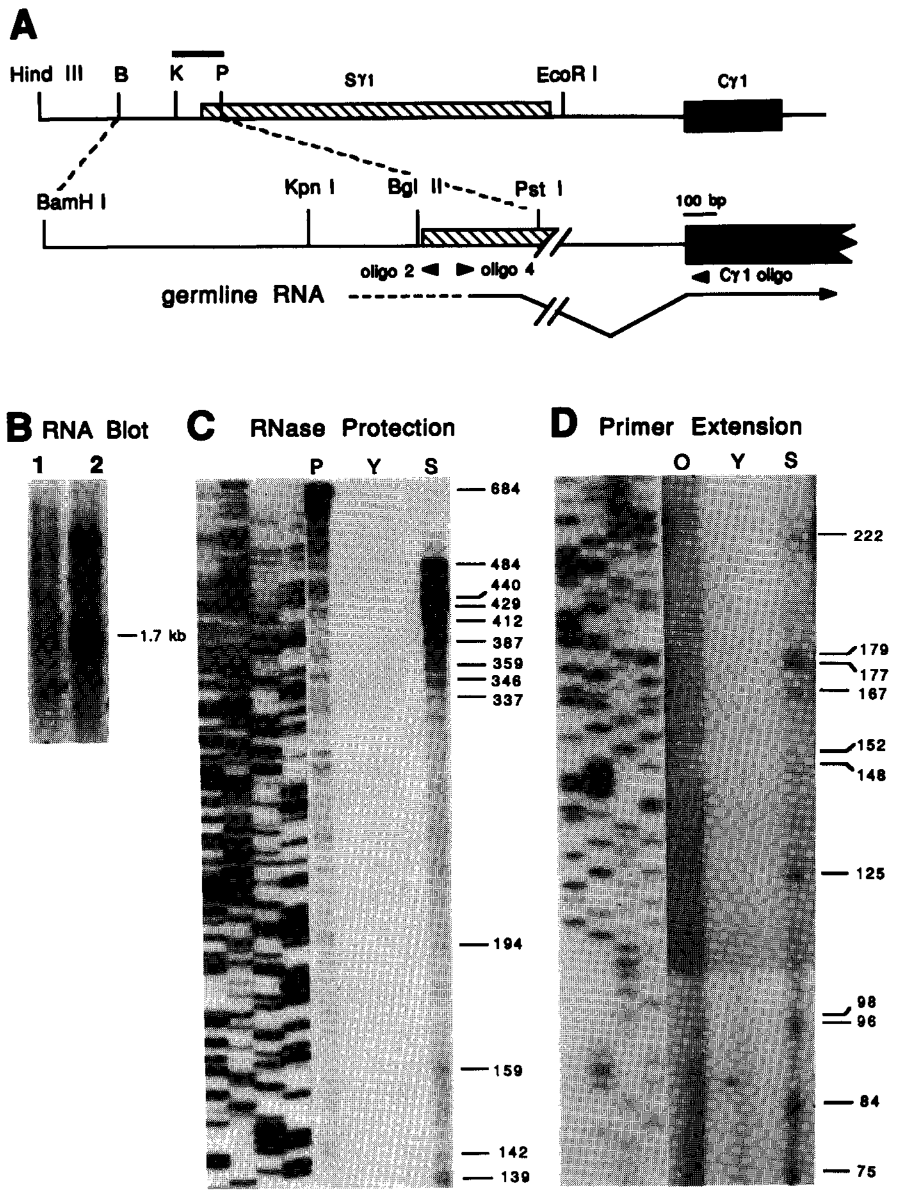

FIGURE 1. (A) Restriction map of the unrearranged genomic $C_{\gamma 1}$ gene. The RNA probe used for hybridization of the RNA blot shown in B and in the RNase protection experiments shown in C is indicated above the maps and the location of oligonucleotides used for primer extension and PCR are shown below the maps. (B) Blot of poly (A)+ RNA (3 μg) from BALB/c spleen cells treated 2 days with LPS (lane 1) or with LPS plus IL-4 (8 U/ml) (lane 2) hybridized with an antisense RNA probe encoding the KpnI-PstI segment. (C) RNase-resistant fragments obtained after hybridization of the RNA probe used in B electrophoresed alongside a DNA-sequencing ladder. Lanes are P, probe alone; Y, probe hybridized with yeast RNA (10 μg); and S, probe hybridized with total cell RNA (10 μg) from spleen (treated as in B). (D) Products of primer-extension experiment using oligo 2 electrophoresed alongside a DNA-sequencing ladder. Lanes are O, oligonucleotide incubated alone; Y, primer extension with yeast RNA (150 μg); and S, primer extension with total cell RNA (150 μg) from spleen (treated as in B).

### Initiation Sites of Germline γ1 RNA

The initiation sites for germline γ1 RNA were determined by RNase-protection and primer-extension experiments. In a RNase-protection experiment, hybridization of RNA from spleen cells (induced with LPS plus 15% HeLa cell supernatant) with a RNA probe transcribed from the genomic DNA KpnI-PstI segment produced multiple bands after electrophoresis of the RNase resistant products on a DNA-sequencing gel (Fig. 1C). The lengths of the predominant bands varied from 387 to 484 nucleotides. Less predominant bands of 139 to 359 nucleotides in length were also observed. These results indicated that the 5′ border of the $I_{\gamma 1}$ exon occurred at multiple sites since we had only found a single 3′ splice site, suggesting that the germline γ1 RNA may have heterogeneous initiation sites. To confirm this and to more precisely locate the initiation sites, primer-extension experiments were performed. As the RNase-protection experiments indicated the major initiation sites are located 5′ of the BglII site, an oligonucleotide (oligo 2) complementary to the sequence from 8–24 nucleotides 3′ to the BglII site

**A**

```
Kpn I
ggtaccgcctcaccctcacccacacattcacatgaagtaatctaagtcaggtttggactccccctcaccctctgacacag   80

aaacccccagaatgaaggggaaccctgtcaggaaatgccttatgccacccactgtcaatcctgttcttaGTCAATCATAT  160

GATGGAAAGAGGGTAGCATTCACCTCTCTGGGACAAAGGCTGTGACTCTGGGAAAGACAAGAGAAGGGCAGGACCAAAAC  240

AGGAACAGAGACGGCTGCTTTCACAGCTTCCACATGTGAGTGGGGTCAGCAGGGAAAGGAGCTGCACGAAGAGGCCATAC  320

                                   Bgl II     oligo 2
AAACAGCACGCATCTGTGGCCCTTCCAGATCTTTGAGTCATCCTATCACGGGAGATTGGGAAGGAGTTGACAGACCAGCC  400

                                                         oligo 4
CAGGCAGTGGAAGCCTCTGTGTGTTAAAGAGTAAAGGTGCTTGCCTACAGCCTGGTGTCAACTAGGCAGGCCCTGGGGGG  480

CGAAGGGGCCTCCTAGACAAGCACAGGCATGTAGAGCTGCACACCCCACAGACAAACCTGAGCCCCGAGGATATCATGGA  560

ATATATCGAGAAGCCTGAGGAATGTGTTTGGCATGGACTACAGGTTGAGAGAACCAAGGAAGCTGAGCCTGCGgtaagtg  640

                                                                       Pst I
accaggggagtgggcggggaggagatatccaagagcattcttaggaatgccttcaagcctgcag                  684
```

**B**

```

C γ1 genomic DNA..................................tatacttttcttgtagCCAAAAC

```

**C**

```

ATGGAATATATCGAGAAGCCTGAGGAATGTGTTTGGCATGGACTACAGGTTGAGAGAACCAAGGAAGCTGAGCCTGCGCC

 m  e  y  i  e  k  p  e  e  c  v  w  h  g  l  q  v  e  r  t  k  e  a  e  p  a  p

                                                C γ1 oligo

AAAACGACACCCCCATCTGTCTATCCACTGGCCCCTGGATCTGCTGCCCAAACTAACTCCATGGTGACCCTGGGATGCCT

  k  r  h  p  h  l  s  i  h  w  p  l  d  l  l  p  k  l  t  p  w ter

```

**D**

```

γ2b RNA { ...........tcaggAAGTGttccg   ....... most 5' initiation site

        { ...........agcagcAGTCttctt   ....... most 3' initiation site

α RNA   { ...........gcgctcAGTCtgacc   ....... first initiation site

        { ...........tcgcccAGTCctaag   ....... second initiation site

γ1 RNA    ...........gttcttAGTCaatca   ....... most 5' initiation site
```

FIGURE 2. (A) DNA sequence of the KpnI-PstI fragment. The sequence was obtained from Genbank (data of Wesley Dunnick) and confirmed by us. Uppercase letters represent $I_{\gamma 1}$ exon and lowercase letters represent the 5′ and 3′ flanking sequences. Turned arrows indicate initiation sites. Triangles indicate the $I_{\gamma 1}/C_{\gamma 1}$ splice site (also in B and C). Sequences present in oligo 2 (used for primer extension) and oligo 4 (used for PCR) are indicated. The initiator Met codon for the potential ORF is boxed. (B) $C_{\gamma 1}$ splice acceptor sequence from Honjo et al. (1979) and found in germline $\gamma 1$ cDNAs produced by the PCR. (C) Open-reading frame (ORF) of the germline $\gamma 1$ RNA. The ORF is initiated in the $I_{\gamma 1}$ exon and terminated within the first exon of $C_{\gamma 1}$. The DNA sequence derived from $C_{\gamma 1}$ is underlined: (D) Comparison of the sequences of the RNA initiation sites of $\gamma 2b$ (Lutzker and Alt, 1988), $\alpha$ (Radcliffe et al., 1990), and $\gamma 1$ RNAs. Thick bars indicate the first nucleotide of these RNAs. The start sites of other $\gamma$, RNAS did not have this sequence.

(Fig. 2A) was used for the primer-extension experiments. The sizes of the primer-extended products (Fig. 1D) matched with those predicted from RNase-protection experiments and indicated that there are multiple initiation sites for germline $\gamma 1$ RNA. The fact that the results from RNase protection and primer extension consistently and completely corresponded indicated that the multiple bands on the gels were not due to the degradation of the RNA. In addition to the predominant initiation sites located 5′ to the BglII site, there are several initiation sites located 3′ to the BglII site that were detected by the RNase protection assay shown in Fig. 1C and by primer-extension experiments (not shown) using an oligonucleotide (oligo 1) complementary to sequences located 75–92 nucleotides 5′ of the splice site. Taken together, the germline $\gamma 1$ RNA has multiple initiation sites distributed over a 345-nucleotide region, but the predominant initiation sites are located within a region of about 97 bp at the 5′ end of the $I_{\gamma 1}$ exon. The most 5′ initiation site of the $I_{\gamma 1}$ exon is 484 bp upstream of the $I_{\gamma 1}/C_{\gamma 1}$ splice site. The sizes of the $I_{\gamma 1}$ exon determined from these experiments (387 to 484 bp) (predominant) and the $C_{\gamma 1}$ exon (1067 bp) (Honjo et al., 1979) would produce a germline $\gamma 1$ RNA of 1.7 kb, assuming a 200-nucleotide poly(A) tail. This corresponds in size to the predominant 1.7-kb germline $\gamma 1$ RNA detected on RNA blots. As is also true for analogous RNAs transcribed from immunoglobulin $\alpha$ (Radcliffe et al., 1990), $\gamma 2b$ (Lutzker and Alt, 1988), and $\mu$ (Lennon and Perry, 1985) genes, there are no TATA or CCAAT boxes located within 150 nucleotides upstream of the initiation sites of the germline $\gamma 1$ RNAs (Fig. 2A).

### The Germline $\gamma 1$ RNA Has an Open-Reading Frame (ORF)

The nucleotide sequence of the $I_{\gamma 1}$ exon indicated the presence of an open-reading frame (ORF) that would be initiated by a Met codon located in such a position that germline $\gamma 1$ RNA initiated at any of the sites we detected would have this Met. Potentially, this ORF would encode a 48 amino acid polypeptide with a termination codon located within the $C_{\gamma 1}$ domain (Figs. 2A and 2C). The reading frame that would be used in the $C_{\gamma 1}$ exon for the ORF in germline RNA differs from that used in the mRNA for $\gamma 1$ H chains. The predicted amino acid sequence of the ORF is indicated below the nucleotide sequence in Fig. 2C. As true for the ORF encoded by germline $\alpha$ RNA, the nucleotide sequence surrounding the initiator AUG codon should form a good translation initiation site according to Kozak (1986, 1987) since it has a purine at the −3 and a G at the +4 positions.

### DISCUSSION

The germline RNA transcribed from unrearranged $C_H$ genes may simply be an indicator of the accessibility of $C_H$ genes. Alternatively, the act of transcription, the RNAs themselves, or their products may function in class switching. The facts that each of the unrearranged $C_H$ genes (except $C_\delta$) have been shown to be transcribed under appropriate conditions (Stavnezer-Nordgren and Sirlin, 1986; Lutzker et al., 1988; Stavnezer et al., 1988; Severinson et al., in press), and that where examined, these transcripts have an I exon located 5′ to the S region, and that transciption proceeds through the S region in the sense direction (Lutzker and Alt, 1988; Stavnezer et al., 1988; Berton et al., 1989; Radcliffe et al., 1990) suggests that this transcription has a function in class switching. Additional common properties are that the germline $\gamma 2b$ (Lutzker and Alt, 1988), $\alpha$ (Radcliffe et al., 1990), $\mu$ (Lennon and Perry, 1985) and $\gamma 1$ RNAs all have multiple initiation sites and no TATA or CCAAT boxes located 5′ of their initiation sites. Some of the initiation sites of $\gamma 2b$, $\mu$, and $\gamma 1$ RNA have similar nucleotide sequences (Fig. 2D), suggesting that they may use a common transcription factor. However, these sequences differ from the initiator sequences that have been defined for other genes that lack CCAAT and TATA boxes (Sehgal et al., 1988; Smale and Baltimore, 1989).

The germline $\gamma 1$ and $\alpha$ RNAs differ from the $\gamma 2b$ and $\mu$ germline RNAs in that the $\gamma 1$ and $\alpha$ transcripts have small ORFs with Met initiation codons in contexts that should allow relatively efficient translation, whereas the $\gamma 2b$ and $\mu$ germline RNAs have small ORFs with Met codons in poor contexts for translation according to Kozak (1986, 1987). The germline $\alpha$ RNA, which has an ORF encoding a 43 amino acid protein (that differs from the sequence encoded by the $\gamma 1$ ORF), appears to be located on small polysomes in I.29$\mu$ B lymphoma cells and this association is enhanced by LPS treatment (Radcliffe et al., 1990). LPS treatment induces class switching from $IgM^+$ to $IgA^+$ in I.29$\mu$ cells (Stavnezer et al., 1985). Thus, it is interesting to speculate that $\alpha$ and

$\gamma 1$ germline RNAs encode polypeptides that function in class switching, but this is probably not the only function of these transcripts, since it appears likely that not all germline RNAs are translated.

The determination of the initiation sites and structure of germline $\gamma 1$ RNA allows us to begin studies to define the DNA regions necessary for regulated expression of the RNAs. It will be important to define these sequences in order to understand how heavy-chain switching is regulated.

### MATERIALS AND METHODS

### Mice and Cell Culture

BALB/c mice were purchased from Charles River Breeding Laboratories (Wilmington, Massachusetts). Spleen cells ($2 \times 10^6$/ml) from mice were cultured for 2 days in RPMI 1640 (GIBCO, Grand Island, New York) in the presence of 10% fetal calf serum (HyClone Laboratories, Logan, Utah). LPS (RIBI Immunochem Research Inc., Hamilton, Montana) was added at 25 $\mu$g/ml. Either 15% HeLa cell supernatant (Bergstedt-Lindqvist et al., 1984), which contains IL-4 and other interleukins, or 8 units/ml of recombinant IL-4 were also added (Noma et al., 1986) (kindly donated by Eva Severinson of the University of Stockholm).

### RNA Isolation and Blot Hybridization

Total cell RNA was prepared by the guanidinium isothiocyanate-CsCl protocol and poly(A)+ RNA was isolated by one cycle of chromatography on oligo(dT)-cellulose. Radioactive RNA probes were transcribed from $5'S_{\gamma 1}$ germline DNA fragments cloned into Bluescript plasmids (Stratagene, La Jolla, California) and hybridization was performed as described (Maniatis et al., 1982).

### DNA Sequencing

Sequenase (United States Biochemicals Corp., Cleveland, Ohio) was used for sequencing plasmid DNA (Tabor and Richardson, 1987).

### Polymerase Chain Reaction (PCR)

PCR was performed according to Frohman et al. (1988). Briefly, 2 $\mu$g of poly(A) + RNA (preheated at 65°C for 3 min and put on ice for 1 min) was reverse transcribed using a $C_{\gamma 1}$ oligo (Figs. 1A and 2C) complementary to $C_{\gamma 1}$ sequence (Honjo et al., 1979) in a 20-$\mu$l reaction containing 50 mM Tris-HCl (pH 8.15), 6 mM $MgCl_2$, 40 mM KCl, 1 mM dithiothreitol (DTT), each dNTP at 1.5 mM, 20 $\mu$Ci of $^{32}$P-dCTP, 25 U of RNase inhibitor (RNasin) (Boehringer Mannheim Biochemicals, Indianapolis, Indiana), and 10 U of avian myeloblastosis virus reverse transcriptase (Life Science, St. Petersburg, Florida) for 2 hr at 42°C. After transcription, the reaction mixture was passed over a 5-ml Sepharose CL-6B column. The first peak (6 drops) was collected and diluted with 500 $\mu$l of 10 mM Tris (pH 8.0), 1 mM EDTA and stored at 4°C. The PCR was performed in 50 $\mu$l of 10% (v/v) dimethyl sulfoxide, 67 mM Tris-HCl (pH 8.8), 16.6 mM $(NH_4)_2SO_4$, 6.7 mM $MgCl_2$, 10 mM 2-mercaptoethanol, 100 $\mu$g/ml BSA, 1.6 mM each dNTP, with 200 ng $C_{\gamma 1}$ oligo, 200 ng oligo 4 (Figs 1A, 2A, and 2C), and 2.5 U of Thermus aquaticus (Taq) DNA polymerase (Perkin-Elmer-Cetus). Just before adding Taq enzyme, the reaction mixture was denatured at 95°C for 5 min and annealed at 45°C for 2 min. 30 $\mu$l of mineral oil (Sigma) was overlaid. The cDNA was amplified at 72°C for 30 min followed by 40 cycle PCR using a Techne programmable Drio-Block machine (American Bioanalytical, Natick, Massachusetts). Each cycle was programmed as 94°C, 1.2 min; 48°C, 2.4 min; and 72°C, 3.6 min. The amplified products were cloned into a Bluescript plasmid with blunt-end ligation and identifield using the $5'S_{\gamma 1}$ BamHI-PstI fragment as a probe (Fig. 1A).

### RNase Protection

Full-length $^{32}$P-labeled antisense RNA probes were transcribed by T7 polymerase from Bluescript plasmids containing various $5'S_{\gamma 1}$ segments. RNase protection analysis was performed using these probes as described (Zinn et al., 1983), except that nuclease P1 (20 $\mu$g/ml) was used instead of RNase A.

### Primer Extension

Primer extension was performed as described (Ausubel et al., 1987). Five ng $^{32}$P-labeled oligo 2 (Figs. 1A and 2A) (labeled using T4 polynucleotide kinase) was mixed with 150 $\mu$g of total cell RNA in 30 $\mu$l of 80% (v/v) deionized formamide, 40 mM PIPES (pH 6.4), 400 mM NaCl, and 1 mM EDTA (pH 8.0), and incubated overnight at 30°C. After ethanol precipitation, 25 $\mu$l of a mixture containing 50 mM Tris-HCl (pH 8.0), 5 mM $MgCl_2$, 5 mM DTT, 50 mM

KCl, 560 $\mu$M of each dNTP, 50 $\mu$g/ml BSA, 1.25 $\mu$l of RNasin, and 40 U reverse transcriptase was added and the reaction was incubated for 90 min at 45°C. 1 $\mu$l of 0.5 M EDTA and 1 $\mu$l of 1 mg/ml RNase A were added and incubation continued for 30 min at 37°C. After phenol/chloroform extraction and ethanol precipitation the extended products were analyzed on 8 M urea sequencing gels.

### ACKNOWLEDGMENTS

This research is supported by a grant, AI23283, from the National Institutes of Health. We thank Dr. Eva Severinson for IL-4 and 2.19 T-cell supernatant.

*(Received September 20, 1989)*

*(Accepted December 5, 1989)*

### REFERENCES

Ausubel F.M., Brent R., Kingston R.E., Moore D.D., Seidman J.G., Smith J.A., and Struhl K., Eds. (1987). Current protocols in molecular biology. (New York: Greene Publishing Associates).

Bergstedt-Lindqvist S., Sideras P., MacDonald H.R., and Severinson E. (1984). Regulation of Ig class secretion by soluble products of certain T-cell lines. Immunol. Rev. **78:** 25.

Bergstedt-Lindqvist S., Moon H-B., Persson U., Moller, G., Heusser C., and Severinson E. (1988). Interleukin 4 instructs uncommitted B lymphocytes to switch to IgG1 and IgE. Eur. J. Immunol. **18:** 1073.

Berton M.T., Uhr J.W., and Vitetta E.S. (1989). Synthesis of germline $\gamma 1$ immunoglobulin heavy-chain transcripts in resting B cells: induction by interleukin 4 and inhibition by interferon $\gamma$. Proc. Natl. Acad. Sci. USA **86:** 2829.

Coffman R.L., Seymour B.W.P., Lebman D.A., Hiraki D.D., Christiansen J.A., Shrader B., Cherwinski H.M., Savelkoul H.F.J., Finkelman F.D., Bond M.W., and Mosmann T.R. (1988). The role of helper T cell products in mouse B cell differentiation and isotype regulation. Immunol. Rev. **102:** 5.

Esser C., and Radbruch A. (1989). Rapid induction of transcription of unrearranged $S_{\gamma 1}$ switch regions in activated murine B cells by interleukin 4. EMBO J. **8:** 483.

Frohman M.A., Dush M.K., and Martin G.R. (1988). Rapid production of full-length cDNAs from rare transcripts: amplification using a single gene-specific oligonucleotide primer. Proc. Natl. Acad. Sci. USA **85:** 8998.

Grimacher C.A. (in press). Molecular aspects of heavy-chain class switching. *In:* CRC Reviews. (Boca Raton, FL: CRC Press).

Honjo T., Obata M., Yamawaki-Kataoka Y., Kataoka T., Kawakami T., Takahashi N., and Mano Y. (1979). Cloning and complete nucleotide sequence of mouse immunoglobulin $\gamma 1$ chain gene. Cell **18:** 559.

Isakson P.C., Pure E., Vitetta E.S., and Krammer P.H. (1982). T cell-derived B cell differentiation factor(s) effect on the isotype switch of murine B cells. J. Exp. Med. **155:** 734.

Kozak M. (1986). Point mutations define a sequence flanking the AUG initiator codon that modulates translation by eukaryotic ribosomes. Cell **44:** 283.

Kozak M. (1987). At least six nucleotides preceding the AUG initiator codon enhance translation in mammalian cells. J. Mol. Biol. **196:** 947.

Lebman D.A., and Coffman R.L. (1988). Interleukin 4 causes isotype switching to IgE in T cell-stimulated clonal B cell culture. J. Exp. Med. **168:** 853.

Lennon G.G., and Perry R.P. (1985). $C_{\mu}$-containing transcripts initiate heterogeneously within the IgH enhancer region and contain a novel 5′-nontranslatable exon. Nature **318:** 475.

Lewin B. (1980). Alternatives for splicing: recognizing the ends of introns. Cell **22:** 324.

Lutzker S., and Alt F.W. (1988). Structure and expression of germ-line immunoglobulin $\gamma 2$b transcripts. Mol. Cell. Biol. **8:** 1849.

Lutzker S., Rothman P., Pollock R., Coffman R., and Alt F.W. (1988). Mitogen- and IL-4-regulated expression of germ-line Ig $\gamma 2$b transcripts: evidence for directed heavy chain class switching. Cell **53:** 177.

Maniatis R., Fritsch E.F., and Sambrook J., Eds. (1982). Molecular cloning: a laboratory manual. (Cold Spring Harbor, NY: Cold Spring Harbor Laboratory Press).

Mowatt M.R., and Dunnick W.A. (1986). DNA sequence of the murine $\gamma 1$ switch segment reveals novel structural elements. J. Immunol. **136:** 2674.

Noma Y., Sideras P., Natio T., Bergstedt-Lindqvist S., Azuma C., Severinson E., Tanabe T., Kinashi T., Matsuda F., Yaoita Y., and Honjo T. (1986). Cloning of cDNA encoding the murine IgG1 induction factor by a novel strategy using SP6 promoter. Nature **319:** 640.

Radcliffe G., Lin Y-C., Julius M., Marcu K.B., and Stavnezer J. (1990). Structure of germline immunoglobulin $\alpha$ heavy-chain RNA and its location on polysomes. Mol. Cell. Biol. 10.

Rothman P., Lutzker S., Cook W., Coffman R., and Alt F.W. (1988). Mitogen plus interleukin 4 induction of $C_{\varepsilon}$ transcripts in B lymphoid cells. J. Exp. Med. **168:** 2385.

Sehgal A., Patil N., and Chao N. (1988). A constitutive promoter directs expression of the nerve growth factor receptor gene. Mol. Cell. Biol. **8:** 3160.

Severinson E., Fernandez C., and Stavnezer J. (1990). Induction of germ line immunoglobulin heavy chain transcripts by mitogens and interleukins prior to switch recombination Europ. J. Immunol. In press.

Smale S.T., and Baltimore D. (1989). The "initiator" as a transcription control element. Cell **57:** 103.

Stavnezer J., Sirlin S., and Abbott J. (1985). Induction of immunoglobulin isotype switching in cultured I.29 B lymphoma cells: characterization of the accompanying rearrangements of heavy chain genes. J. Exp. Med. **161:** 577.

Stavnezer J., Radcliffe G., Lin Y-C., Nietupski J., Berggren L., Sitia R., and Severinson E. (1988). Immunoglobulin heavy-chain switching may be directed by prior induction of transcripts from constant-region genes. Proc. Natl. Acad. Sci. USA **85:** 7704.

Stavnezer-Nordgren J., and Sirlin S. (1986). Specificity of immunoglobulin heavy chain switch correlates with activity of germline heavy chain genes prior to switching. EMBO J. **5:** 95.

Tabor S., and Richardson C.C. (1987). DNA sequence analysis with a modified bacteriophage T7 DNA polymerase. Proc. Natl. Acad. Sci. USA **84:** 4767.

Yancopoulos G.D., DePinho R.A., Zimmerman K.A., Lutzker K., Rosenberg N., and Alt F. W. (1986). Secondary genomic rearrangement events in pre-B cells: $V_HDJ_H$ replacement by a LINE-1 sequence and directed class switching. EMBO J. **5:** 3259.

Zinn K., DiMaio D., and Maniatis T. (1983). Identification of two distinct regulatory regions adjacent to the human $\beta$-interferon gene. Cell **34:** 865.

